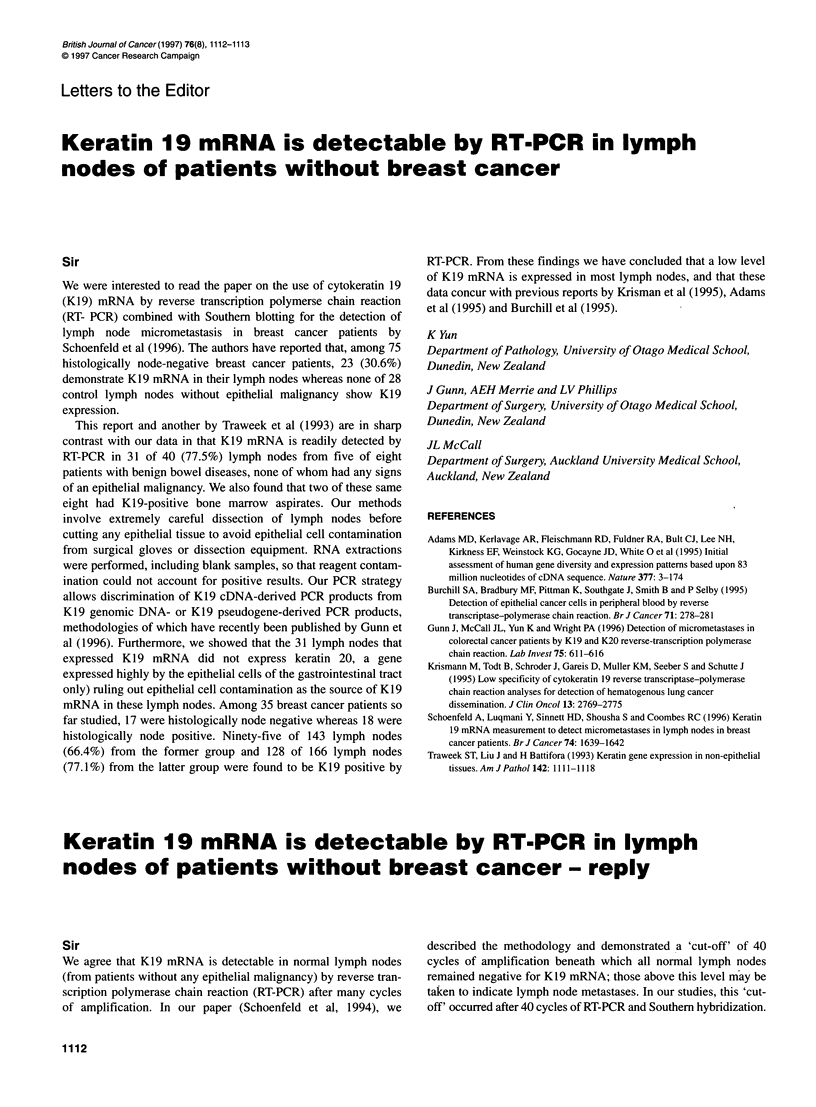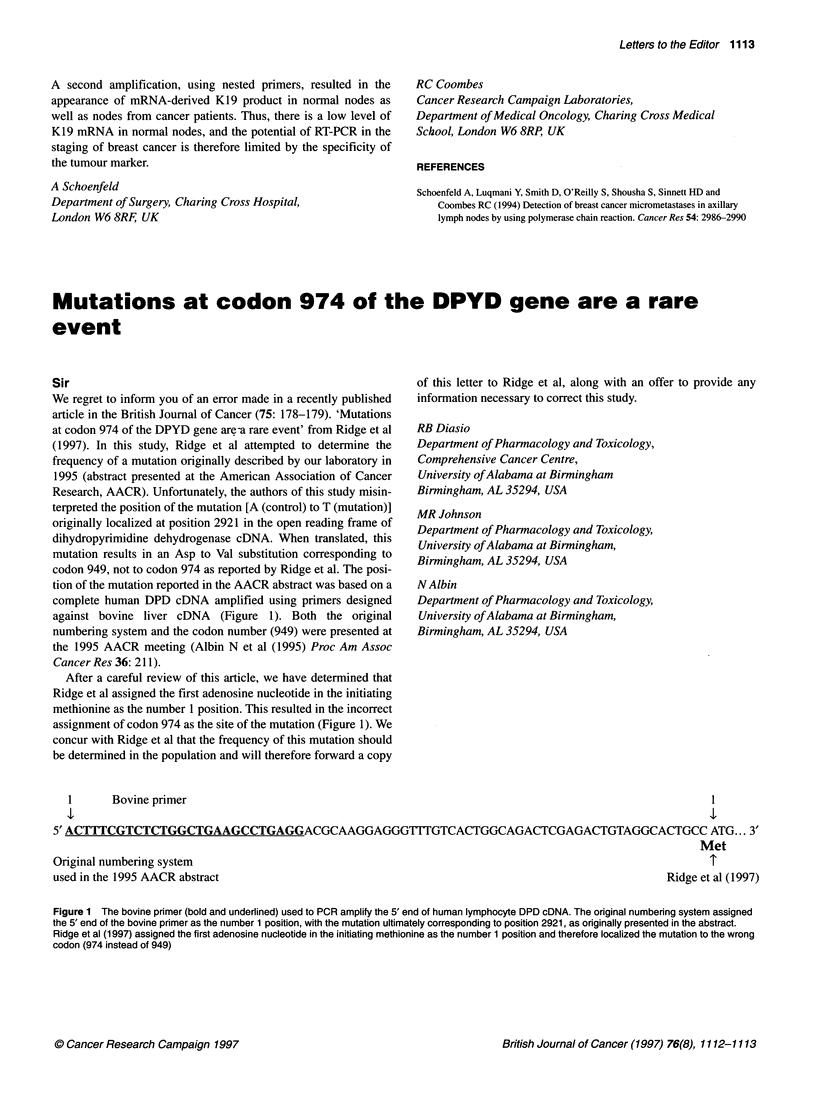# Keratin 19 mRNA is detectable by RT-PCR in lymph nodes of patients without breast cancer-reply

**Published:** 1997

**Authors:** A Schoenfeld, RC Coombes


Letters to the Editor

### Keratin 19 mRNA is detectable by RT-PCR in lymph nodes of patients without breast cancer

### Keratin 19 mRNA is detectable by RT-PCR in lymph nodes of patients without breast cancer – reply

**Sir**

We agree that K19 mRNA is detectable in normal lymph nodes (from patients without any epithelial malignancy) by reverse transcription polymerase chain reaction (RT-PCR) after many cycles of amplification. In our paper (Schoenfeld et al, 1994), we described the methodology and demonstrated a 'cut-off' of 40 cycles of amplification beneath which all normal lymph nodes remained negative for K19 mRNA; those above this level may be taken to indicate lymph node metastases. In our studies, this 'cut-off' occurred after 40 cycles of RT-PCR and Southern hybridization.

A second amplification, using nested primers, resulted in the appearance of mRNA-derived K19 product in normal nodes as well as nodes from cancer patients. Thus, there is a low level of K19 mRNA in normal nodes, and the potential of RT-PCR in the staging of breast cancer is therefore limited by the specificity of the tumour marker.

*A Schoenfeld*
*Department of Surgery, Charing Cross Hospital, London W6 8RF, UK*

*RC Coombes*
*Cancer Research Campaign Laboratories, Department of Medical Oncology, Charing Cross Medical School, London W6 8RP, UK*

**REFERENCES**

Schoenfeld A, Luqmani Y, Smith D, O'Reilly S, Shousha S, Sinnett HD and Coombes RC (1994) Detection of breast cancer micrometastases in axillary lymph nodes by using polymerase chain reaction. *Cancer Res* **54**: 2986–2990